# Compressed sensing acceleration of radial 3‐D alternating Look‐Locker $T_{1}$ mapping

**DOI:** 10.1002/mrm.30610

**Published:** 2025-06-16

**Authors:** Antti Aarnio, Olli Nykänen, Ville Kolehmainen, Mikko J. Nissi

**Affiliations:** ^1^ Department of Technical Physics University of Eastern Finland Kuopio Finland

**Keywords:** accelerated, alternating Look‐Locker, compressed sensing, $T_{1}$ relaxation

## Abstract

**Purpose:**

To determine how various compressed sensing (CS) models can accelerate alternating Look‐Locker $T_{1}$ mapping.

**Methods:**

An alternating Look‐Locker acquisition was retrospectively accelerated by factors of 1–12. The data was reconstructed into 12 images with multiple CS models, which utilized combinations of spatial total variation, locally low‐rank regularization, and subspace constraints. Complex non‐linear least squares signal fitting was performed to obtain the $T_{1}$ maps. The accelerated $T_{1}$ maps were compared against the $T_{1}$ map of a full data reference reconstruction.

**Results:**

A subspace‐constrained reconstruction model with spatial total variation and locally low‐rank regularization outperformed all other models as measured by $T_{1}$ map normalized root mean squared error, structural similarity index, and normalized mean absolute deviation. The subspace constraint benefited models utilizing spatial total variation but, conversely, did not benefit models utilizing only locally low‐rank regularization.

**Conclusion:**

The radial 3‐D alternating Look‐Locker $T_{1}$ mapping acquisition was successfully accelerated by up to a factor of 12 with various CS models. The best‐performing model was a subspace‐constrained reconstruction, which utilized spatial total variation and locally low‐rank regularization.

## 1 INTRODUCTION

Recently, an alternating Look‐Locker (aLL) acquisition^1^ was proposed for simultaneous $T_{1\rho }$ and $T_{1}$ mapping. As a $T_{1}$ mapping method, aLL is robust against radio frequency field inhomogeneities^1^, a clear benefit over variable flip angle methods, and accounts for an imperfect inversion^1^ offering more accurate $T_{1}$ estimates^2^ compared to traditional Look‐Locker methods.^3^, ^4^ The downside of the aLL acquisition, and most quantitative MRI (qMRI), is that the acquisition is relatively long.

Compressed Sensing (CS)^5^, ^6^, ^7^ is a mathematical theory that can be leveraged for accelerated MRI.^8^ Model‐based,^9^, ^10^, ^11^, ^12^ subspace‐constrained,^13^, ^14^, ^15^, ^16^ and standard CS approaches^17^ have been applied for the acceleration of $T_{1}$ mapping methods. Model‐based approaches solve the non‐linear signal model and usually yield the highest accelerations, but also suffer from high computational cost, limiting their feasibility for non‐Cartesian 3‐D qMRI. Subspace‐constrained approaches leverage the signal model, but alleviate the computational burden by approximating it with a low‐dimensional subspace.^18^, ^19^ Standard CS methods ignore the signal model and promote some general forms of sparsity instead.

The performance of a CS model is related to the acquisition itself. The object of interest affects the appropriate form of spatial sparsity, signal evolution over the acquired image series relates to temporal sparsity, and the k‐space sampling trajectories determine the outlook of the undersampling artifacts.^8^ In our previous work, a combination of spatial total variation and locally low‐rank regularization was shown to be the best‐performing model for radial 3‐D variable flip angle $T_{1}$ mapping.^17^

In this work, the acceleration of radial 3‐D aLL $T_{1}$ mapping with standard and subspace‐constrained CS models is studied. The aim was to determine, in a radial 3‐D qMRI setting, the achievable acceleration and to assess the effect of the subspace constraint. Based on the previous work,^13^, ^14^, ^16^, ^17^ the tested CS models utilized combinations of a subspace constraint,^20^, ^21^, ^22^, ^23^, ^24^ spatial total variation,^25^ and locally low‐rank^26^ regularizations. Tiny golden means^27^ was adopted as the sampling strategy as it promises approximately uniform sampling for any amount of data.

## 2 THEORY

### 2.1 Alternating Look‐Locker

The aLL acquisition^1^ comprises of two sequentially alternating parts. In the first part ($S^{-}$), after a signal saturation and a fixed relaxation delay, the magnetization is inverted, and continuous signal sampling is performed until the signal reaches steady state. In the second part ($S^{+}$), the same procedure is repeated but without the inversion pulse. After binning the acquired data to a desired number of k‐spaces, the aLL signal model can be written as

(1) $$\begin{array}{cc} S^{-}(t_{i}) & =M_{\text{ss}}-(M_{\text{ss}}+IM_{0})\exp(t_{i}/T_{\text{eff}}),\\ S^{+}(t_{i}) & =M_{\text{ss}}-(M_{\text{ss}}-M_{0})\exp(t_{i}/T_{\text{eff}}), \end{array}$$

where $t_{i}$ denotes the time points of the binned images, $M_{\text{ss}}$ is the steady state magnetization, $M_{0}$ is the magnetization recovered after the saturation and relaxation delay, I is a parameter that accounts for an imperfect inversion, and $T_{\text{eff}}$ is the effective relaxation time.^1^

The Equation (1) provides estimates for the $M_{\text{ss}}$, $M_{0}$, I, and $T_{\text{eff}}$ values. The $T_{1}$ is then estimated from the $M_{0}$/$M_{\text{ss}}$ ratio^1^

(2) $$T_{1}(1-\exp(-T_{d}/T_{1}))=\frac{M_{0}T_{R}}{M_{\text{ss}}(1-\exp(-T_{R}/T_{\text{eff}}))},$$

where $T_{d}$ is the relaxation delay, and $T_{R}$ is the repetition time.

### 2.2 Image reconstruction

The MRI forward model for the multi‐image aLL acquisition can be written as

(3) y=Ex+w,

where $x\in {\mathbb{C}}^{TN}$ contains the whole image series, $y\in {\mathbb{C}}^{TM}$ is the binned k‐space data, E is a block diagonal matrix that contains all the forward operators mapping the data from image domain to k‐space, $w\in {\mathbb{C}}^{TM}$ models the complex Gaussian measurement noise, T is the number of images, N is the number of voxels in an image, and M is the number of acquired datapoints. If parallel imaging is utilized,^28^ coil sensitivities are incorporated into the forward operator.

If the Nyquist sampling criterion is approximately fulfilled, the standard least‐squares (LS) approach can be used to estimate the images, i.e.,

(4) $$\hat{x}=\arg\underset{x}{\min}\frac{1}{2}\|Ex-y{\|}_{2}^{2},$$

where $\hat{x}$ is the image series estimate. With radial acquisitions, an isotropic resolution image with N voxels requires $\pi {\left(\sqrt[{3}]{N}\right)}^{2}$ uniformly distributed spokes.^29^

#### 2.2.1 Compressed sensing

CS can be used to solve problems where the Nyquist criterion is violated, i.e., where the acquisition is undersampled. The prerequisite for CS is that the undersampling yields noise‐like artifacts which can be efficiently eliminated by promoting some form of sparsity in the solution.^5^, ^8^ As such, CS models extend the LS model with sparsity‐promoting regularizations, yielding

(5) $$\hat{x}=\arg\underset{x}{\min}\frac{1}{2}\|Ex-y{\|}_{2}^{2}+\underset{r}{\sum }\alpha_{r}R_{r}(x),$$

where $R_{r}$ are the regularization terms and $\alpha_{r}$ the corresponding regularization parameters. The regularization parameter controls the weighting between the data fidelity term and the regularization functionals.

#### 2.2.2 Subspace‐constrained reconstruction

The subspace‐constrained reconstruction utilizes prior information about the acquisition to learn a low‐dimensional temporal basis in which the image reconstruction is performed.^13^, ^15^, ^16^, ^20^, ^30^ Subspace‐constrained reconstruction is

(6) $$\hat{\lambda }=\arg\underset{\lambda }{\min}\frac{1}{2}\|E\Phi_{K}\lambda -y{\|}_{2}^{2}+\underset{r}{\sum }\alpha_{r}R_{r}(\lambda ),$$

where $\lambda \in {\mathbb{C}}^{KN}$ contains the subspace coefficient images, and $\Phi_{K}\in {\mathbb{C}}^{TN\times KN}$ implements the temporal subspace basis. The image domain image series can be recovered from the subspace coefficient images after the reconstruction with $\hat{x}=\Phi_{K}\hat{\lambda }$. More specifically, for a voxel r, the temporal signal evolution is represented by a linear combination of basis vectors as $\Phi_{K}\lambda (r)=\sum_{k=1}^{K}\lambda_{k}(r)u_{k}$ where $u_{k}$ is the k‐th basis vector and $\lambda_{k}(r)$ is the estimated coefficient. The temporal subspace basis can be estimated, e.g., with principal component analysis from a series of simulated signals.^18^, ^19^, ^20^

#### 2.2.3 Regularizations

Spatial total variation (STV) assumes that the spatial image gradient is sparse and promotes piece‐wise constant images.^8^, ^25^ The isotropic STV can be written as

(7) $$R_{r}(u)={\left\|\sqrt{{\left(\nabla_{x}u\right)}^{2}+{\left(\nabla_{y}u\right)}^{2}+{\left(\nabla_{z}u\right)}^{2}}\right\|}_{1},$$

where ∇ implements the first‐order finite differences for the subscript dimension.

Locally low‐rank (LLR) regularization^26^ promotes sparsity of the singular value spectrum of a local image region. It has been applied in various qMRI applications and shown to outperform the globally low‐rank model.^17^, ^30^, ^31^ The LLR regularization can be presented as

(8) $$R_{r}(u)=\underset{b\in \Omega }{\sum }\|C_{b}(u){\|}_{*},$$

where $C_{b}$ is an operator that extracts a local region b from the image series and reshapes it to a Casorati form,^26^, ^31^ Ω is the set of all local image regions and $\|\cdot {\|}_{*}$ denotes the nuclear norm. Each column of the Casorati matrix is a vectorized representation of the local image region, and the number of columns is equal to the number of acquired images.^22^

## 3 METHODS

### 3.1 Data acquisition

The 3 h 30 min aLL Multi‐Band‐SWIFT acquisition^1^, ^32^ was performed with a 9.4T Varian/Agilent MRI scanner (VnmrJ DirectDrive software v3.2, Varian Associates Inc., Palo Alto, CA, USA) and a 19 mm quadrature radiofrequency volume transceiver (Rapid Biomedical, Rimpar, Germany). Previously prepared ex vivo rat brain fixed in 4% paraformaldehyde was immersed in perfluoropolyether (Galden HS240, Vacuumservice Oy, Helsinki, Finland) and imaged. MB‐SWIFT parameters were: 192 kHz bandwidth, 4° flip angle, 3.2 cm isotropic field of view, 256^3^ image size, 2.9624 ms repetition time, twice oversampled readouts, 4 gaps, and 128 sidebands. For aLL, the signal inversion was performed with a hyperbolic secant pulse^33^ (HS4, R20, 3ms, 2.5 kHz) and saturation with an adiabatic half passage pulse^34^ (3ms, 2.5 kHz). The relaxation delay was 3 s. The relaxation was sampled over 1 476 spokes and binned to 6 images. The time points $t_{i}$ of the images were 360, 1 090, 1 820, 2 550, 3 280, 4 010 ms.

For the k‐space trajectory, 2 510 299 order‐55 tiny golden angle (tGA)^27^ spokes were generated and partitioned into 12 sections. Additional spokes were linearly interpolated between consecutive tGA spokes to ensure that the acquisition trajectory, obtained by inverse sorting the 12 k‐spaces, does not contain large jumps between consecutive spokes. A total of 2 532 816 unique spokes were acquired.

### 3.2 Reconstruction

Eight different reconstruction models were tested (Table 1). The models were compared against a reference reconstruction in a region of interest covering the whole specimen with normalized root mean squared error (nRMSE), structural similarity index (SSIM),^35^ and median normalized absolute deviation (MNAD).

**TABLE 1:** The tested image reconstruction models, their acronyms, and regularizations terms.

Acronym	Model (eq.)	Regularization (eq.)
Reference	(5)	(8)
STV	(5)	(7)
S‐STV	(6)	(7)
LLR	(5)	(8)
S‐LLR	(6)	(8)
STV+LLR	(5)	(7) and (8)
S‐STV+LLR	(6)	(7) and (8)
LS	(4)	—
S‐LS	(6)	—

*Note*: The CS models utilized a combination of a subspace constraint (S), isotropic spatial total variation (STV), or locally low‐rank (LLR) regularization. Least squares (LS) reconstruction models, which utilize no regularization, were also tested. The LLR block sizes were $13^{3}$ for the reference and $8^{3}$ for all other models, and non‐overlapping blocks were utilized.

Acceleration factors (AF, the amount of data required for theoretical full sampling divided by the amount used in the reconstruction) of 1, 3, 6, 9, and 12 were tested. For the reference reconstruction, AF=0.975. The reference reconstruction regularization strength was chosen based on a visual inspection of the $T_{1}$ map such that the noise was minimized but no image blurring or visible blocks were introduced. For other regularized models, the minimum $T_{1}$ nMRSE solution was used unless regularization artifacts were visible. In such cases, the regularization parameter was reduced until no excessive artifacts remained.

Alternating direction method of multipliers (ADMM)^36^, ^37^ was used to solve all regularized image reconstruction models. Preconditioned LSMR^38^, ^39^, ^40^ was used to solve the non‐regularized reconstruction models and the least‐squares step of the ADMM. For ADMM, 30 ADMM iterations and 2 inner LSMR iterations with warm start were used. LSMR reconstructions used 5 iterations. The forward operator was approximated with a non‐uniform fast Fourier transform.^41^, ^42^ All algorithms were implemented in Python with JAX.^43^

For the subspace generation, the $T_{\text{eff}}$, $M_{\text{ss}}$, $M_{0}$, and I parameter ranges were determined by evaluating histograms of the reference reconstruction parameter values in a region of interest covering the whole specimen. The subspace dictionary was generated with Equation (1) by linearly choosing 401 $T_{\text{eff}}$ values from 0.5 to 0.9 s, 17 $M_{\text{ss}}$ values from $0.74M_{0}$ to $0.9M_{0}$, and 78 I values from 0.23 to 1.0 resulting in 122 706 signal representations. The dictionary was compressed with singular value decomposition to K=4 components, to yield below 1% maximum signal‐wise nRMSE for the subspace.

Parameter maps were estimated with complex non‐linear signal fitting^44^, ^45^, ^46^ and a bisection method.^47^ Following,^1^ a 3‐D Gaussian filter with a standard deviation of 0.6 and a radius of 4 standard deviations, both in voxels, was used on all reconstructed images before the signal fitting to mitigate some residual image noise.

## 4 RESULTS

**FIGURE 1:**
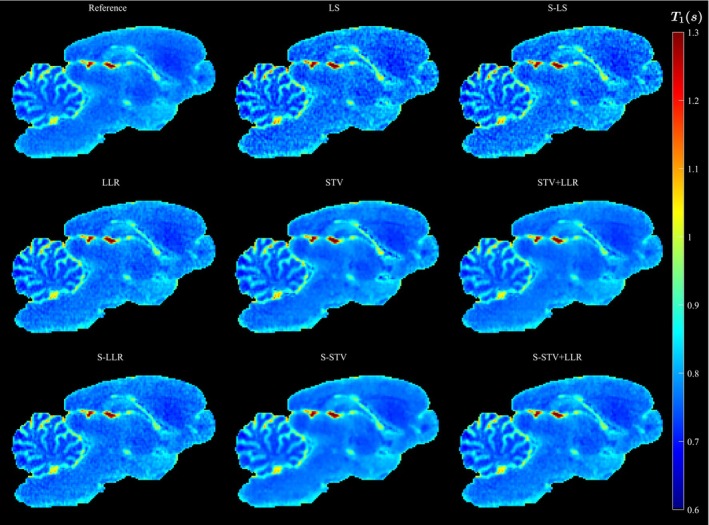
Example image slice of the 3‐D $T_{1}$ map for all reconstruction models and AF=1.

The $T_{1}$ maps for AF=1 demonstrate how well the chosen models resemble the reference (Figures 1 and S1 & S2). The reference reconstruction is considerably less noisy than the LS and S‐LS because of the regularization. LLR and S‐LLR resemble the reference reconstruction well. The STV and S‐STV $T_{1}$ maps have overall a smoother appearance, with the former generally being noisier. The STV+LLR and S‐STV+LLR retain more noise compared to the STV and S‐STV but are less noisy than the LLR and S‐LLR models.

**FIGURE 2:**
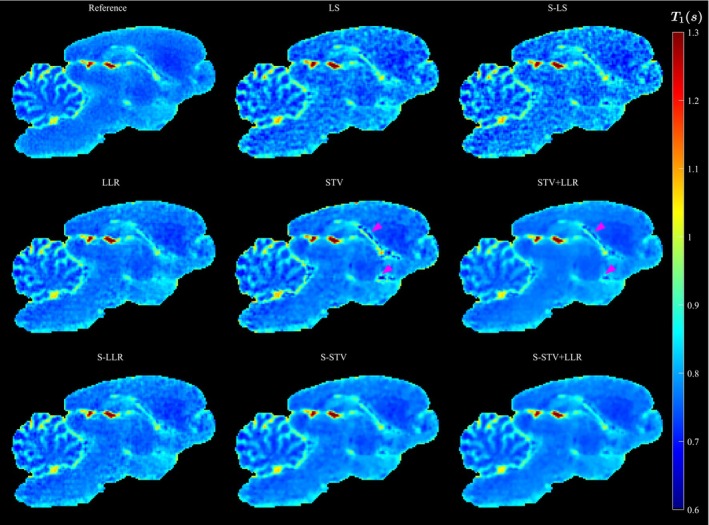
Example image slice of the 3‐D $T_{1}$ map for all reconstruction models and AF=6. The arrowheads point to example regions where the STV and STV+LLR erroneously introduce signal voids.

For AF=6, the S‐STV+LLR outperforms other models as contrast around different tissues is maintained around the cerebellum, and noise is efficiently minimized. The subspace constraint improves the $T_{1}$ map for STV models, as more details in the cerebellum are retained and less noise and staircasing artifacts are present, but essentially no benefit is observed for LLR‐only models. The STV and STV‐LLR models introduce signal voids in the $T_{1}$ map (Figure 2 arrows), which are not observed in the subspace‐constrained model versions. The LS and S‐LS models suffer from considerable noise (Figures 2 and S5 & S6).

**FIGURE 3:**
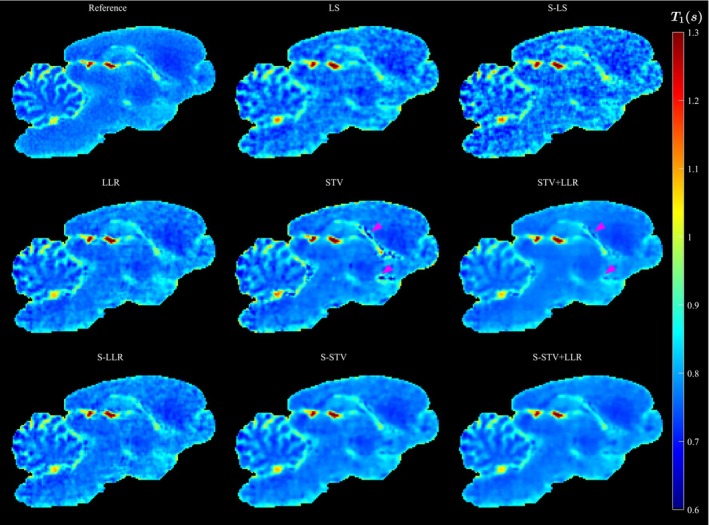
Example image slice of the 3‐D $T_{1}$ map for all reconstruction models and AF=12. The arrowheads point to example regions where the STV and STV+LLR erroneously introduce signal voids.

Small image details and contrast between tissues are diminished with the highest acceleration of AF=12 (Figures 3 and S5 & S6). Otherwise, similar observations can be made as in the AF=6 case. Subspace constraint only benefits models utilizing STV, and S‐STV+LLR is the best‐performing model. Signal voids are again present for STV and STV+LLR.

**FIGURE 4:**
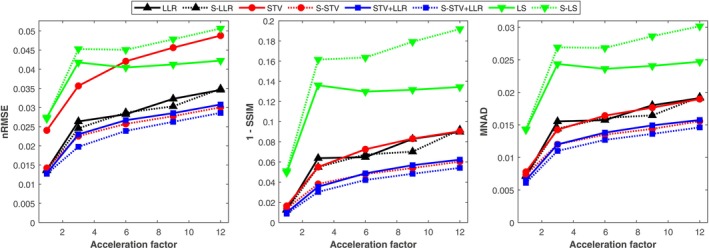
Normalized root mean squared error (nRMSE), structural similarity index (SSIM) represented in 1‐SSIM scale to aid visual interpretation with the other metrics, and median normalized absolute deviation (MNAD) as a function of the acceleration factor for the tested CS models. The error metrics were calculated against the reference reconstruction in a region of interest covering the whole brain.

The $T_{1}$ nRMSE, SSIM, and MNAD support the previous observations (Figure 4). The error metrics agree with each other, and the best‐performing model is the S‐STV+LLR. The subspace constraint benefits models utilizing STV but generally does not benefit the LLR‐only model.

## 5 DISCUSSION

Subspace‐constrained model utilizing spatial total variation and locally low‐rank regularization, S‐STV+LLR, was the best reconstruction model. The S‐STV‐LLR model was also the most sophisticated model and required tuning of two regularization parameters and choosing the appropriate subspace. While the subspace parameters can be determined from, e.g., previous scans or literature, tuning of the regularization parameters without a fully sampled reference remains an open problem. Some methods have been proposed for automatic regularization tuning,^48^, ^49^, ^50^, ^51^ but they have limited use cases. The S‐STV model had nearly the same performance but with one less regularization parameter. Careful consideration should thus be given when choosing the appropriate reconstruction model.

The subspace constraint significantly improved spatial total variation, while locally low‐rank regularization performed similarly with or without the constraint. Since the subspace is derived from principal component analysis, the first coefficient image contains most of the relevant information and has the highest SNR, with the SNR and information content of subsequent coefficient images rapidly decreasing. Thus, while locally low‐rank regularization yields higher‐quality, low‐information‐content coefficient images, their contributions to the original image series are minimal. Similarly, spatial total variation benefits from the subspace because small image details can be better distinguished from noise in the first coefficient image, which has high SNR and information content. This trend likely generalizes to other spatial and temporal regularizations as well.

For the generation of the subspace, the quantitative parameter ranges were chosen to be as small as possible. This allows for the subspace to be compressed as efficiently as possible, but might be an unreasonable approach in practice since the exact ranges of the parameters may not be known in advance. Larger dictionaries might necessitate the use of a larger subspace to maintain appropriate modeling error. Increasing the subspace size lowers the modeling error but also amplifies the reconstruction noise.^30^ Modifying the subspace size possibly affects the results.

Usually, some form of phase correction is utilized with total variation since image phase can cause the real and imaginary components to not be piecewise constant even if the magnitude image itself is.^8^, ^52^ MB‐SWIFT images have relatively constant minimal phase, since essentially no phase evolution happens during imaging,^32^ which does not significantly affect the total variation regularization. If parallel imaging is utilized, the phase of the radiofrequency coil elements will affect the image phase and possibly deteriorate the performance of the spatial total variation if no phase correction is utilized.

The aLL acquisition utilized parameters from the original publication.^1^ However, CS reconstruction can be expected to change the optimal values for some of these parameters. For example, the lower SNR of a smaller flip angle acquisition can be partially resolved with regularization. Smaller flip angle in turn would increase the scanning efficiency as the $T_{\text{eff}}$ is lengthened, and a larger proportion of the scan time would be spent sampling instead of waiting. The aLL acquisition would also allow for simultaneous $T_{1\rho }$ mapping by adding an appropriate $T_{1\rho }$ magnetization preparation block into the acquisition,^1^ which is an obvious benefit of the aLL method. The addition of the $T_{1\rho }$ preparation block introduces $T_{1\rho }$ relaxation contributions to the $T_{\text{eff}}$ values. Essentially, this would be visible as a modified contrast between tissues in the image series, which could affect the performance of the CS models. Optimization of the acquisition parameters in combination with a CS reconstruction, specifically for the combined acquisition of $T_{1}$ and $T_{1\rho }$, is an interesting direction for future studies.

The scan time for the aLL acquisition of this study was 3.5 h. However, for clinical scanners, a faster realization of the aLL sequence can be achieved by leveraging parallel imaging methods^28^ and, e.g., a zero echo time (ZTE) readout.^53^ By a conservative estimate, parallel imaging can provide a two‐fold acceleration and can easily be incorporated into the reconstruction models. Additionally, since the acquisition used does not require the use of MB‐SWIFT, the change from 4 times gapped MB‐SWIFT to ZTE readout would provide an additional four‐fold acceleration. With the ZTE substitution, the SNR is, however, reduced, as the inherent averaging of MB‐SWIFT is lost,^32^ which might affect the results.

Notably, there is significant interest in deep learning methods for MR image or quantitative parameter reconstruction. A 3‐D non‐Cartesian quantitative MRI acquisition, however, presents challenges for the deep learning methods because the dimensionality of the data is large, and additional measures are required to take into account the non‐Cartesian nature of the data. At the moment, radial 3‐D qMRI applications also do not have significant amounts of training data available for DL development. A problem, which is highlighted even more for studies on preclinical magnets, where the measurement setup and the object of interest can be quite unique. Thus, deep learning was not utilized in this study.

The largest limitation of this study was the LLR regularized reference reconstruction and the manual tuning of the regularization parameters. Given the lack of true ground truth, the reference reconstruction should have a high SNR to minimize possible error stemming from the noise. Acquiring more data would have been the most correct approach, but was infeasible due to the already long acquisition time. Thus, an LLR regularization approach was chosen instead. With minimal regularization, in theory, only singular value components corresponding to pure noise are eliminated with LLR. LLR regularization also provides clear visual guidelines for the manual regularization tuning. High regularization introduces image blur and block‐like artifacts, and low regularization retains most of the noise. Possible bias was minimized by utilizing different LLR block sizes for the reference and other reconstructions. Given that the S‐STV model was one of the best‐performing models and the LLR models did not clearly outperform others, we believe that the regularized reference did not significantly bias the results. The manual tuning of the regularization parameter was performed to eliminate visible over‐regularization artifacts from the minimum error $T_{1}$ maps of the LLR, S‐LLR, STV, and S‐STV models and introduces some subjectivity to the results.

## 6 CONCLUSIONS

The 3‐D aLL $T_{1}$ mapping was successfully accelerated utilizing various CS models. The best‐performing model was a subspace‐constrained reconstruction, which utilized spatial total variation and locally low‐rank regularization. Reconstruction models utilizing spatial total variation benefited from the subspace constraint.

## CONFLICT OF INTEREST STATEMENT

The authors declare no potential conflict of interest.

## Supporting information


**Figure S1:** Example image slice of the 3‐D $T_{1}$ map for all reconstruction models and AF = 1. The $T_{1}$ image stack was rotated using bilinear interpolation and re‐sliced to acquire the slices shown, which slightly affects the visual outlook.
**Figure S2:** Example image slice of the 3‐D $T_{1}$ map for all reconstruction models and AF = 1. The $T_{1}$ image stack was rotated using bilinear interpolation and re‐sliced to acquire the slices shown, which slightly affects the visual outlook.
**Figure S3:** Example image slice of the 3‐D $T_{1}$ map for all reconstruction models and AF = 6. The $T_{1}$ image stack was rotated using bilinear interpolation and re‐sliced to acquire the slices shown, which slightly affects the visual outlook.
**Figure S4:** Example image slice of the 3‐D $T_{1}$ map for all reconstruction models and AF = 6. The $T_{1}$ image stack was rotated using bilinear interpolation and re‐sliced to acquire the slices shown, which slightly affects the visual outlook.
**Figure S5:** Example image slice of the 3‐D $T_{1}$ map for all reconstruction models and AF = 12. The $T_{1}$ image stack was rotated using bilinear interpolation and re‐sliced to acquire the slices shown, which slightly affects the visual outlook.
**Figure S6:** Example image slice of the 3‐D $T_{1}$ map for all reconstruction models and AF = 12. The $T_{1}$ image stack was rotated using bilinear interpolation and re‐sliced to acquire the slices shown, which slightly affects the visual outlook.
