# Supplementary material for: Compressed sensing acceleration of radial 3‐D alternating Look‐Locker T1 mapping
Source: Magn Reson Med. 2025 Jun 16;94(5):2258–67. doi: 10.1002/mrm.30610 (PMC12393196; doi:10.1002/mrm.30610)
Supplement: Supplementary file 1 — Figure S1: Example image slice of the 3‐D T1 map for all reconstruction models and AF = 1. The T1 image stack was rotated using bilinear interpolation and re‐sliced to acquire the slices shown, which slightly affects the visual outlook. Figure S2: Example image slice of the 3‐D T1 map for all reconstruction models and AF = 1. The T1 image stack was rotated using bilinear interpolation and re‐sliced to acquire the slices shown, which slightly affects the visual outlook. Figure S3: Example image slice of the 3‐D T1 map for all reconstruction models and AF = 6. The T1 image stack was rotated using bilinear interpolation and re‐sliced to acquire the slices shown, which slightly affects the visual outlook. Figure S4: Example image slice of the 3‐D T1 map for all reconstruction models and AF = 6. The T1 image stack was rotated using bilinear interpolation and re‐sliced to acquire the slices shown, which slightly affects the visual outlook. Figure S5: Example image slice of the 3‐D T1 map for all reconstruction models and AF = 12. The T1 image stack was rotated using bilinear interpolation and re‐sliced to acquire the slices shown, which slightly affects the visual outlook. Figure S6: Example image slice of the 3‐D T1 map for all reconstruction models and AF = 12. The T1 image stack was rotated using bilinear interpolation and re‐sliced to acquire the slices shown, which slightly affects the visual outlook. [file MRM-94-2258-s001.pdf]

Compressed sensing acceleration of radial 3-D alternating Look-Locker T_1 mapping

Antti Aarnio[1] Olli Nykänen[1] Ville Kolehmainen[1]
Mikko J. Nissi[1]

[1] Department of Technical Physics, University of Eastern Finland

Supplementary material

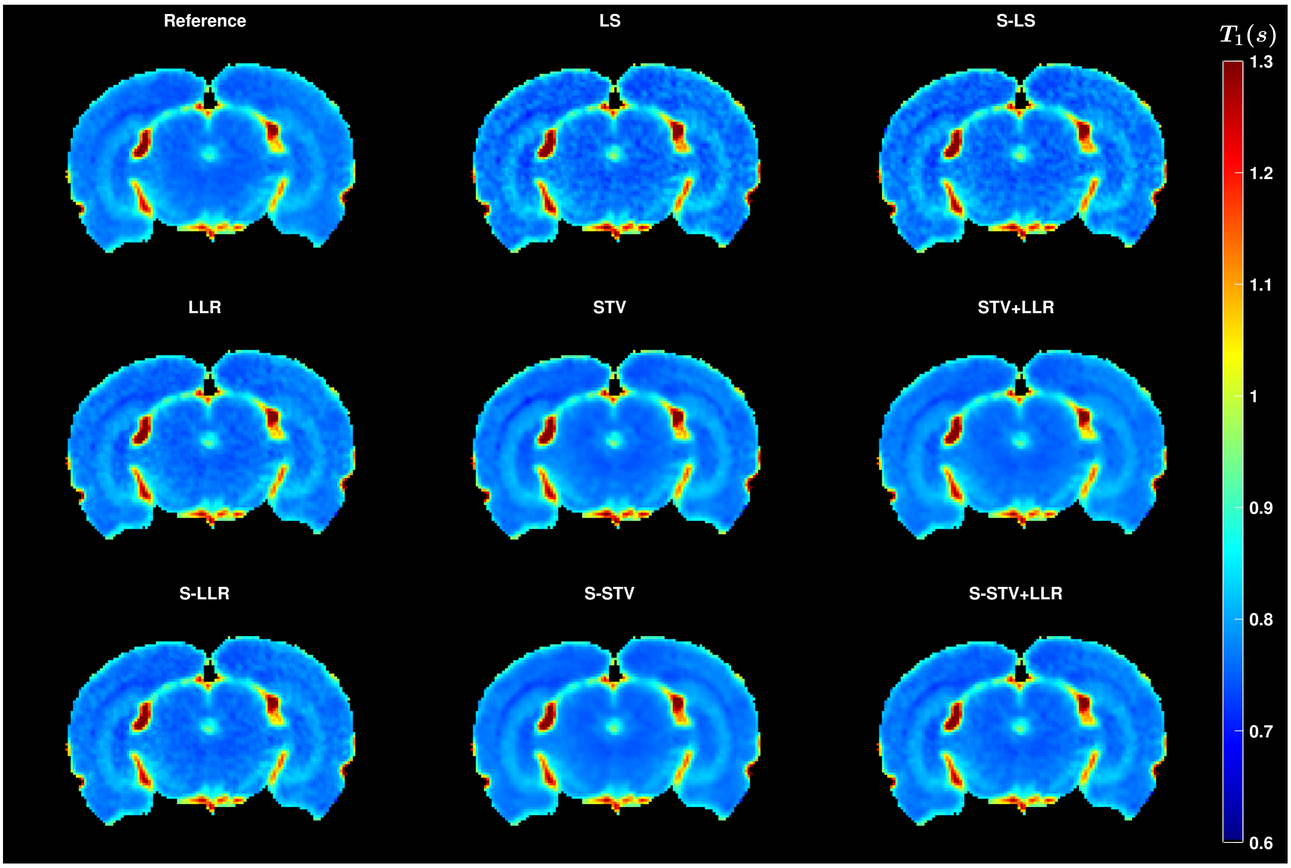

Figure S1: Example image slice of the 3-D T_1 map for all reconstruction models and AF = 1. The T_1 image stack was rotated using bilinear interpolation and re-sliced to acquire the slices shown, which slightly affects the visual outlook.

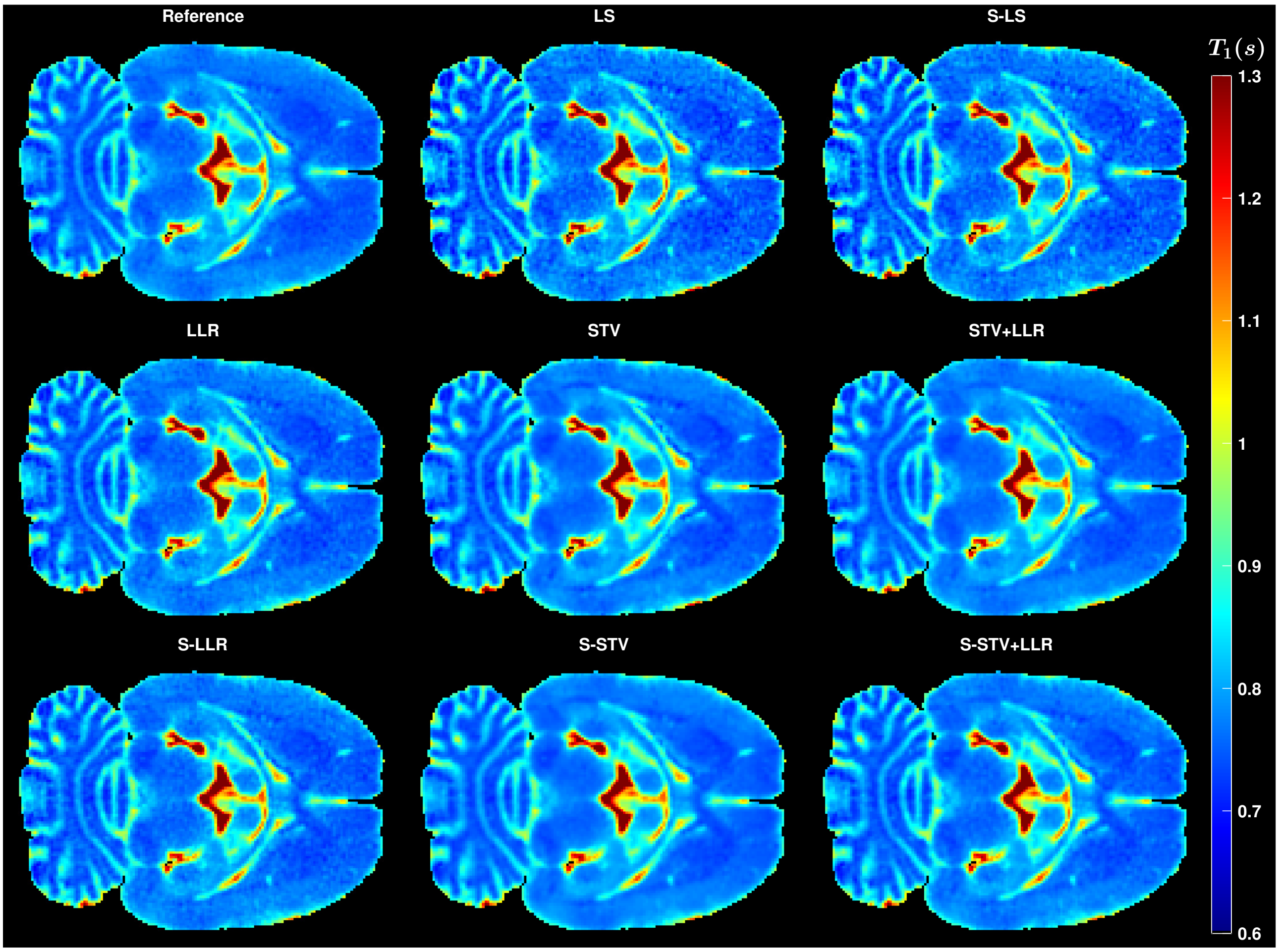

Figure S2: Example image slice of the 3-D T_1 map for all reconstruction models and AF = 1. The T_1 image stack was rotated using bilinear interpolation and re-sliced to acquire the slices shown, which slightly affects the visual outlook.

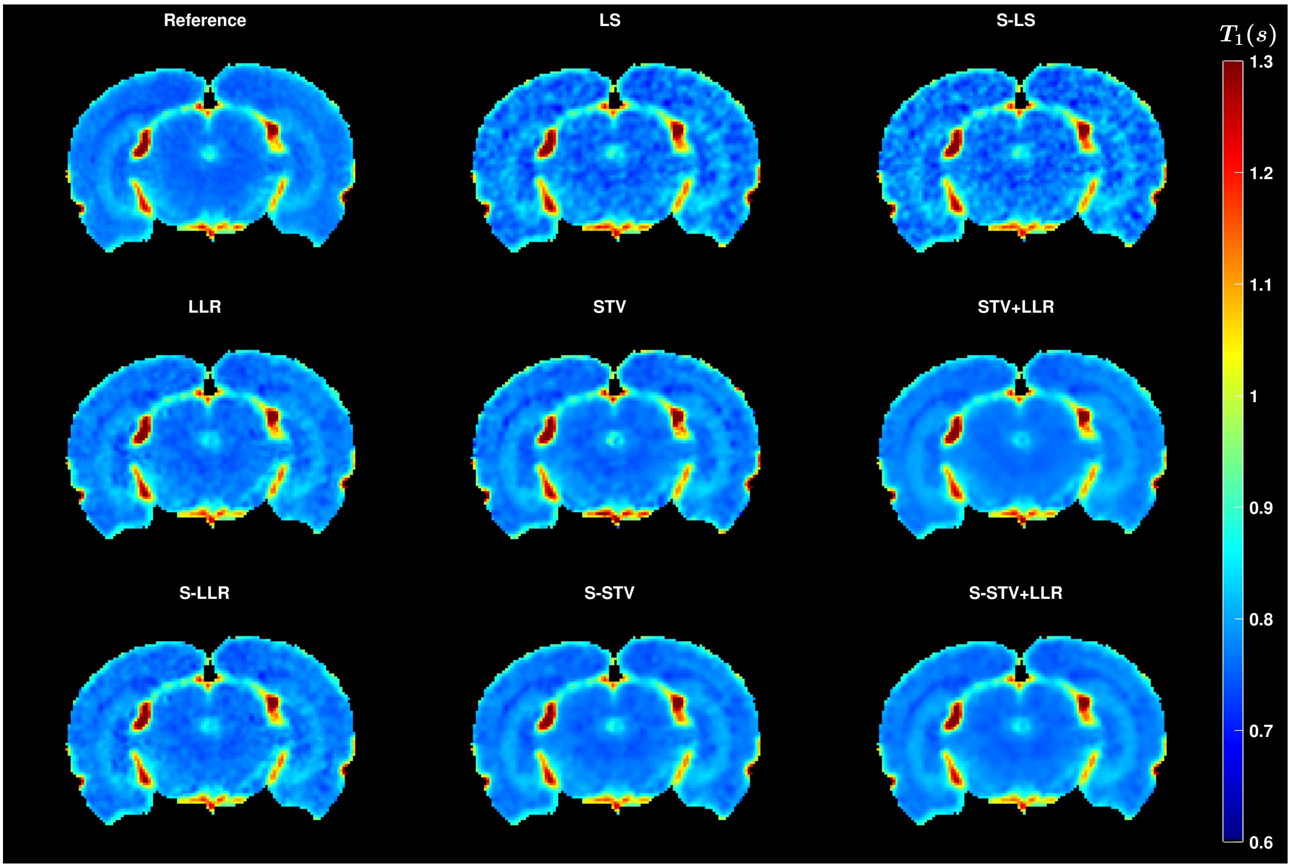

Figure S3: Example image slice of the 3-D T_1 map for all reconstruction models and AF = 6. The T_1 image stack was rotated using bilinear interpolation and re-sliced to acquire the slices shown, which slightly affects the visual outlook.

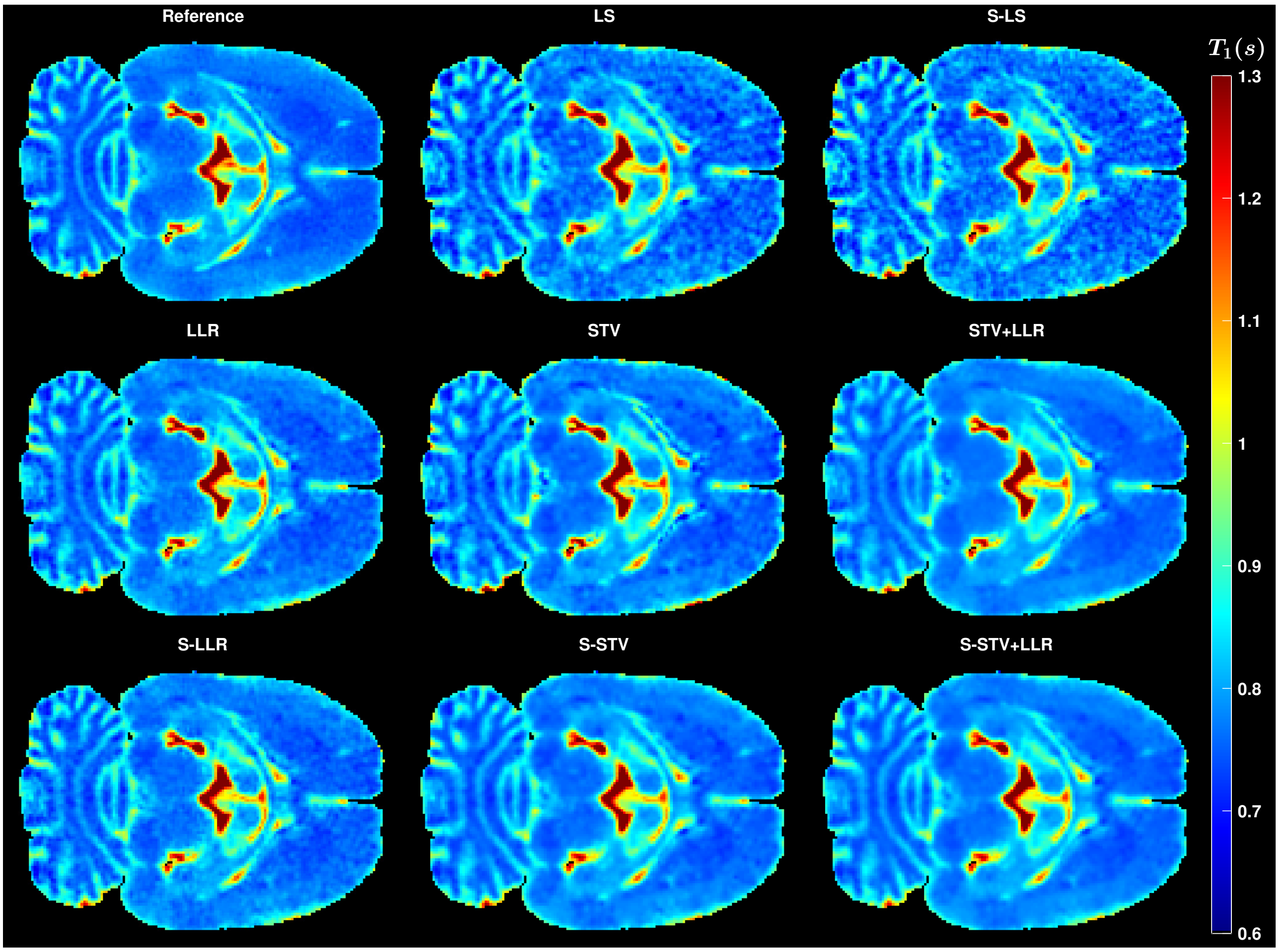

Figure S4: Example image slice of the 3-D T_1 map for all reconstruction models and AF = 6. The T_1 image stack was rotated using bilinear interpolation and re-sliced to acquire the slices shown, which slightly affects the visual outlook.

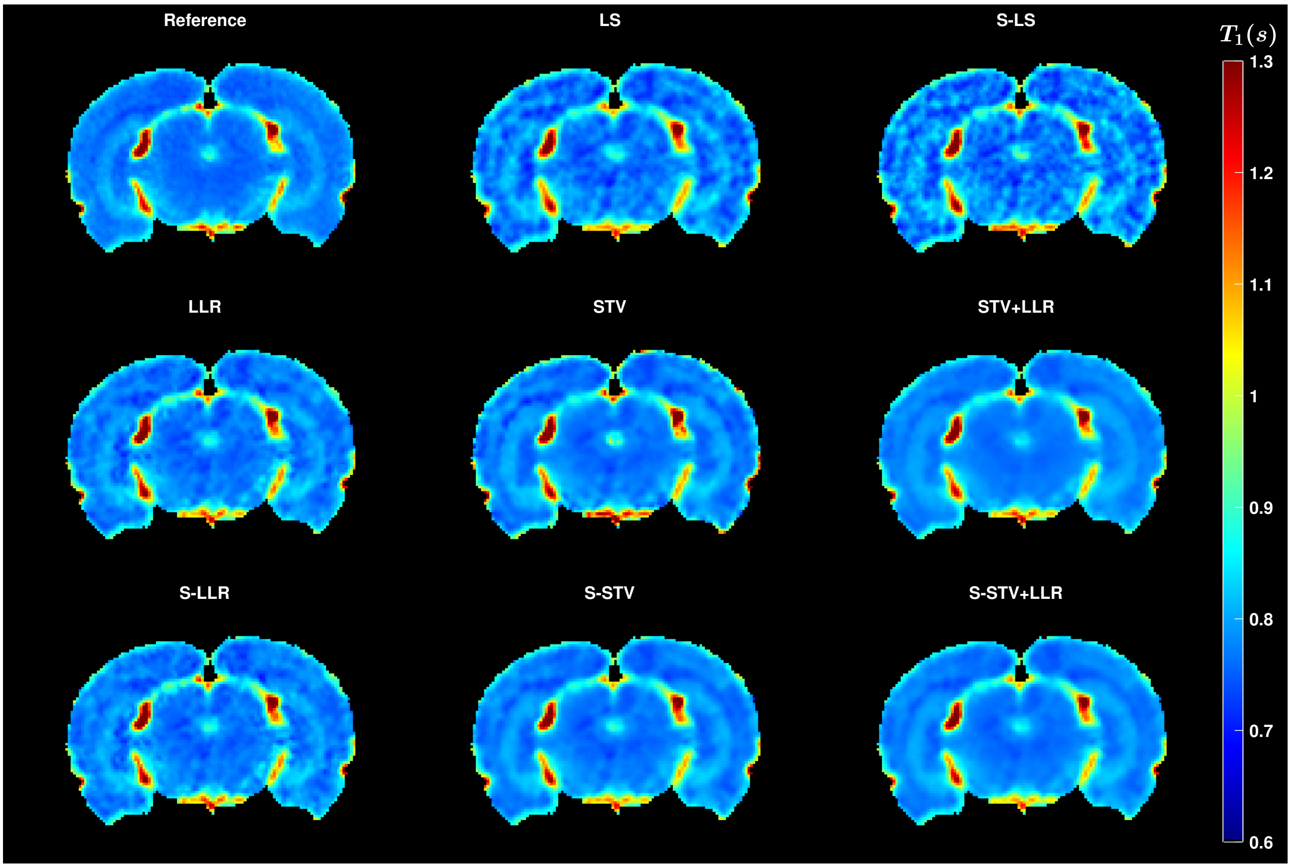

Figure S5: Example image slice of the 3-D T_1 map for all reconstruction models and AF = 12. The T_1 image stack was rotated using bilinear interpolation and re-sliced to acquire the slices shown, which slightly affects the visual outlook.

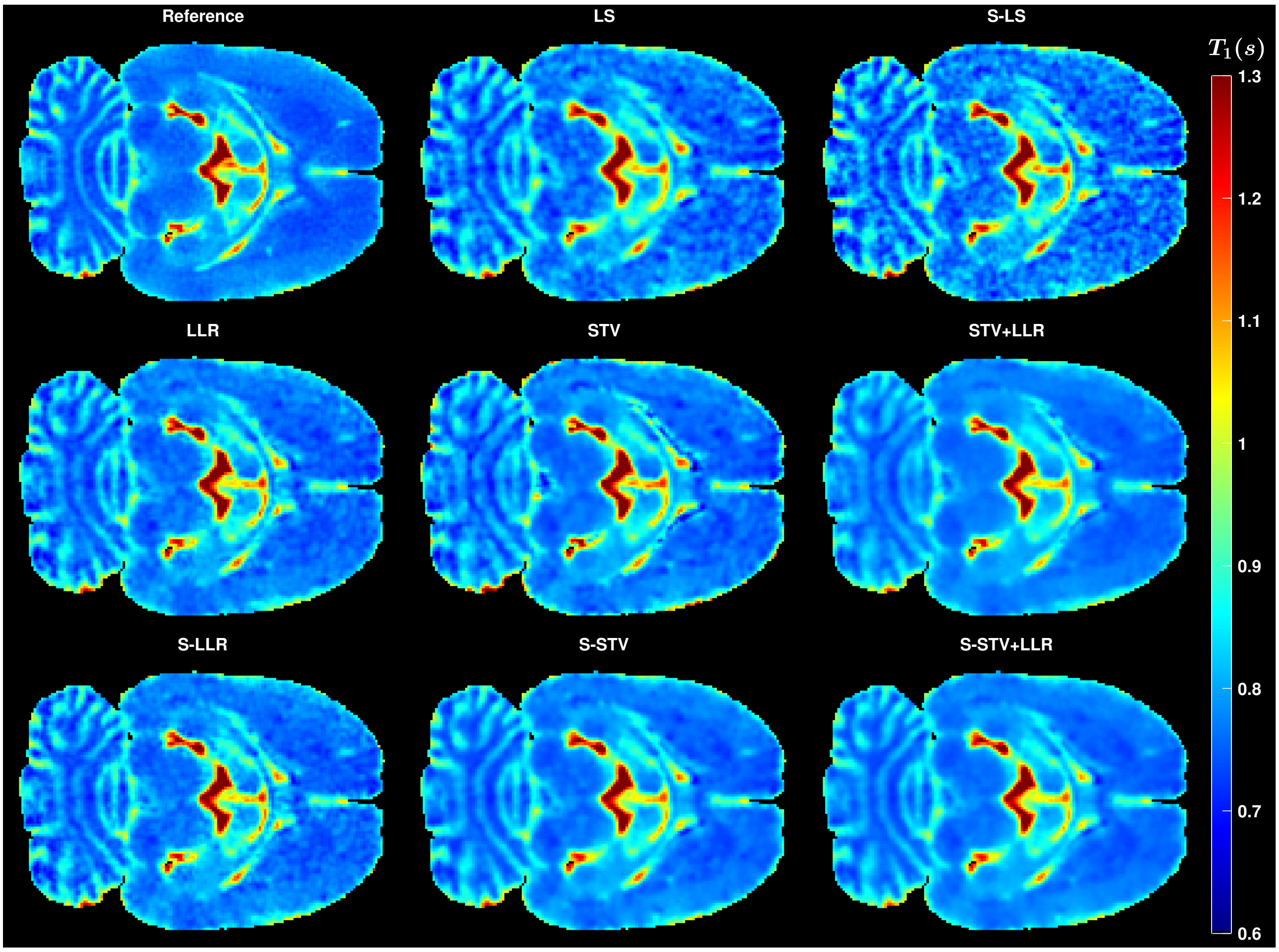

Figure S6: Example image slice of the 3-D T_1 map for all reconstruction models and AF = 12. The T_1 image stack was rotated using bilinear interpolation and re-sliced to acquire the slices shown, which slightly affects the visual outlook.
